# Fusion of Environmental Sensing on PM_2.5_ and Deep Learning on Vehicle Detecting for Acquiring Roadside PM_2.5_ Concentration Increments

**DOI:** 10.3390/s20174679

**Published:** 2020-08-19

**Authors:** Wen-Cheng Vincent Wang, Tai-Hung Lin, Chun-Hu Liu, Chih-Wen Su, Shih-Chun Candice Lung

**Affiliations:** 1 Research Center for Environmental Changes, Academia Sinica, Nangang, Taipei 115, Taiwan; phdzen@gate.sinica.edu.tw (W.-C.V.W.); lch0909@gate.sinica.edu.tw (C.-H.L.); 2 Department of Information & Computer Engineering, Chung Yuan Christian University, Taoyuan 320, Taiwan; jacklin@cycu.org.tw; 3 Department of Atmospheric Sciences, National Taiwan University, Taipei 106, Taiwan; 4 Institute of Environmental Health, National Taiwan University, Taipei 106, Taiwan

**Keywords:** air pollution sensing, low-cost sensor, particle source, transportation emission, vehicle counting, vehicle classification

## Abstract

Traffic emission is one of the major contributors to urban PM_2.5_, an important environmental health hazard. Estimating roadside PM_2.5_ concentration increments (above background levels) due to vehicles would assist in understanding pedestrians’ actual exposures. This work combines PM_2.5_ sensing and vehicle detecting to acquire roadside PM_2.5_ concentration increments due to vehicles. An automatic traffic analysis system (YOLOv3-tiny-3l) was applied to simultaneously detect and track vehicles with deep learning and traditional optical flow techniques, respectively, from governmental cameras that have low resolutions of only 352 × 240 pixels. Evaluation with 20% of the 2439 manually labeled images from 23 cameras showed that this system has 87% and 84% of the precision and recall rates, respectively, for five types of vehicles, namely, sedan, motorcycle, bus, truck, and trailer. By fusing the research-grade observations from PM_2.5_ sensors installed at two roadside locations with vehicle counts from the nearby governmental cameras analyzed by YOLOv3-tiny-3l, roadside PM_2.5_ concentration increments due to on-road sedans were estimated to be 0.0027–0.0050 µg/m^3^. This practical and low-cost method can be further applied in other countries to assess the impacts of vehicles on roadside PM_2.5_ concentrations.

## 1. Introduction

Sensing technology for environmental pollutants has been rapidly developing in recent years [1,2]. The application of low-cost sensors (LCSs) provides opportunities to tackle research challenges that have been difficult to address before [3,4]. The targeted environmental pollutant in this work is particulate matter with an aerodynamic diameter less than or equal to 2.5 μm (PM_2.5_), which is a classified human carcinogen [5] with annual mean levels of up to 100 μg/m^3^ in many urban areas around the world [6,7], much higher than 10 μg/m^3^, the annual recommended guideline of the World Health Organization [8]. The Global Burden of Disease Study 2015 showed that around 5.7–7.3 million deaths could be attributable to PM_2.5_ [9,10]. For urban PM_2.5_, traffic is the single largest contributor, accounting for 25% globally [11]. Accurately estimating on-road vehicle emissions for PM_2.5_ could reduce uncertainty in current PM_2.5_ source apportionment and formulate effective control measures to lower PM_2.5_ levels and associated health risks [12].

In addition, pedestrians walking in streets may be exposed to direct vehicle emissions, resulting in higher PM_2.5_ exposures than the ambient levels [13]. Scientifically, accurately assessing human exposure leads to more precise estimates for damage coefficients of exposure–health relationships [14]. Exposure levels can be estimated as the sum of ambient levels (background levels not affected by nearby sources) and exposure increments, which come from different nearby sources. Ambient levels of PM_2.5_ concentrations are regularly measured at the regulatory monitoring stations of Taiwan Environmental Protection Administrations (EPA). Nevertheless, very few studies have assessed roadside PM_2.5_ exposure levels [15,16] that occur in close proximity to pedestrians who suffered from peak levels higher than those assessed in the regulatory monitoring stations of EPAs worldwide [13], which are typically situated at 10–15 m above ground. This gap might be filled by the newly developed LCSs.

LCS devices are devices that integrate LCS, power, and data transmission components. Several PM_2.5_ LCSs have been shown to meet the precision criteria of the United States Environmental Protection Agency (USEPA) for continuous PM_2.5_ monitoring (r > 0.9 or R^2^ > 0.81; [17]) or candidate equivalent methods (r > 0.97 or R^2^ > 0.94; [18]) [19,20,21]. After data correction with equations obtained from side-by-side comparison with research-grade instruments, the research-grade-comparable measurements from these LCS devices can be used for environmental research. One of these PM_2.5_ LCS devices is AS-LUNG-O; its readings could be converted to research-grade- or Federal-equivalent-method (FEM)-comparable measurements and used for community source evaluations [13,21]. AS-LUNG-O was used in this work to assess PM_2.5_ levels at roadsides.

In our previous study [13], traffic contributions to PM_2.5_ increments can be estimated based on the measurements of LCSs. However, PM_2.5_ increments were the overall contributions of all vehicle counts. The contribution of “each” vehicle was unknown. With vehicle counting in this work, the contribution of each vehicle can be calculated. Therefore, we applied an automatic traffic analysis system in order to extract useful traffic information from different closed-circuit television (CCTV) scenes simultaneously. Innovative techniques of deep learning have been developed for object detection in recent years [22]. This work applies deep learning and the traditional optical flow technique for detecting and tracking vehicles, respectively, to obtain traffic counts of different vehicle types in real time. This is particularly useful in countries with huge numbers of motorcycles, which have been difficult to count with traditional methods that target four-wheeled vehicles. Through data fusion of traffic counts of different vehicle types from this innovative traffic analysis system and PM_2.5_ levels from LCS devices, roadside PM_2.5_ concentration increments due to vehicles can be acquired accordingly.

The objectives of this work are to (1) evaluate an automatic traffic analysis system based on deep learning and optical flow techniques, (2) apply the automatic traffic analysis system in the field along with PM_2.5_ sensing, and (3) acquire roadside PM_2.5_ concentration increments due to vehicles with data fusion of PM_2.5_ sensing and vehicle counting. By analyzing 488 images from 23 governmental CCTV cameras, the automatic traffic analysis system was found to have an 87% precision rate and 84% recall rate. By combining PM_2.5_ sensing and vehicle counting at two roadside locations, we successfully demonstrate a methodology to estimate roadside PM_2.5_ concentration increments due to vehicles. This innovative and relatively low-cost methodology can be applied in other countries, especially low- and middle -income countries, to estimate pedestrians’ PM_2.5_ exposure increments at street levels due to vehicles.

## 2. Materials and Methods

This section presents a combination of the automatic traffic analysis system, real-time measurements from LCSs, and the statistical regression model to evaluate roadside PM_2.5_ concentration increments.

### 2.1. Vehicle Classification and Counting Method

At present, the object detection methods based on deep learning are basically divided into two categories: two-stage and one-stage object detectors. The general processing flow of the two-stage detector was described in [22]. Faster regions with convolutional neural networks (Faster R-CNN) [23] is a classic two-stage approach, which evolved from R-CNN [24] and Fast R-CNN [25]. It extracts the feature map of the potential object region through the region proposal network (RPN). In the second stage, it corrects the region proposals to locate objects more accurately and applies object classification to them. Although the two-stage detector is regarded as more accurate than the one-stage detector, the detection speed is limited by the classification stage. There are too many object proposals to be classified, so it is not suitable for real-time application. A standard Faster R-CNN can only achieve five frames per second (fps) performance. Therefore, the idea of the one-stage detector has been developed [22]. The aim of single-stage detection is to predict the object location and category without the second step refinement. A single neural network predicts object positions and types directly from an input image in one evaluation, thereby reducing the time consumption. You Only Look Once (YOLO) [26] is one famous example. It takes color images as network input and divides the image into N × N grid cells. Each grid cell predicts B bounding boxes and confidence scores for those boxes. This method is fast in detection (45 fps), but due to the lack of object positioning, the detection accuracy is not high enough, and it is also inefficient in predicting tiny objects. The subsequent version, YOLOv2 [27], improves the accuracy by adding some techniques such as batch normalization, convolutional with anchor boxes, multi-scale training, and change structure of feature extractor (Darknet-19). It is indeed a breakthrough in detection, but it still cannot deal with the small object problem. In 2018, YOLOv3 [28] was proposed and considered one of the state-of-the-art one-stage object detection methods. It includes a feature pyramid network (FPN) and residual network to increase the detection ability of small objects and to generate a deeper network architecture, respectively. YOLOv3 can achieve 20 fps, which is much faster than Faster R-CNN and other one-stage detectors, as summarized in [27].

Although the detection accuracy of YOLOv3 is high enough, the inference time and model size are not acceptable for analyzing multiple streaming videos in practice. In general, a real-time object detector should process 30 fps or higher, but YOLOv3 cannot achieve this requirement on a consumer-grade graphic processing unit (GPU). Furthermore, in terms of model size, YOLOv3 requires 1.6 GB GPU memory, which is still large for analyzing multiple CCTV videos concurrently with a consumer-grade GPU. In this case, a top-class GPU (e.g., Nvidia RTX 2080ti with 11 GB memory) only supports six video inputs at the same time. If the model size can be halved, twice the number of video inputs can be supported. As a result, we chose YOLOv3-tiny-3l [29] as an alternative. YOLOv3-tiny-3l is a modified lightweight version of YOLOv3 that reduces some convolutional layers in the network but preserves the FPN structure. The simplified network can efficiently detect objects without reducing the accuracy too much and can ensure that the GPU memory consumption only costs about 0.8 GB.

This work acquired videos from governmental cameras and conducted on-line streaming analysis in real time whenever possible. If videos could not be accessed on-line, they were saved on disks regularly from the governmental computers and analyzed afterwards. Vehicle classification and counting were carried out with YOLOv3-tiny-3l processed in an Intel Core i7-7800X computer environment with XPG Z1 DDR4 3000 16G × 2 RAM, MSI GeForce GTX1080Ti graphic cards with 11G memory × 2, MSI X299 GAMING PRO CARBON mainboard, Micron 1TB SSD, and the Ubuntu 18.04 system.

Just recently, a new breakthrough in one-stage object detectors, YOLOv4, made its debut [30]. The authors disassembled the CNN object detection architecture into four parts: input, backbone, neck, and head. They optimized some of these parts for higher detection accuracy and speed. The experiments show that it improves YOLOv3′s average precision and FPS by 10% and 12%, respectively. Such a fast and accurate object detector no longer requires high-end graphic cards (Tesla V100) for training. Instead, it can be easily trained using ordinary consumer graphic cards (GTX1080ti/RTX2008ti). This type of object detector is suitable for individuals or small research teams to develop and use. As a result, we evaluated the performance of the YOLOv4 model for different input sizes in comparison with YOLOv3-tiny-3l.

### 2.2. Performance Evaluation of YOLOv3-Tiny-3l

**Figure 1:**
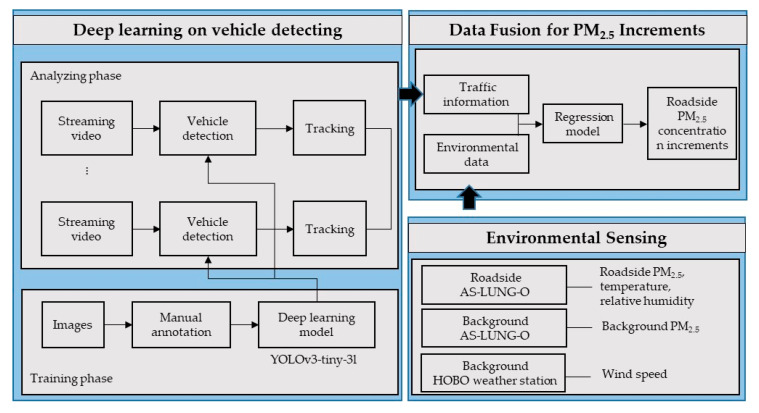
The system flowchart of the data flow from both vehicle-detecting and PM_2.5_ sensing to data fusion for PM_2.5_ increments.

The application of YOLOv3-tiny-3l is shown in the system flowchart as two phases: training and analyzing (Figure 1). To train a reliable and robust detector, it is important to get a representative and sufficient amount of training data for manual labeling beforehand. Therefore, collecting traffic images that contain different vehicle appearances that are as varied as possible from different CCTV cameras is required. In the training phase, we manually labeled 2439 images from 23 governmental CCTV cameras in Taiwan, which were set up by transportation authorities to monitor traffic congestion or accidents. Unfortunately, the governmental CCTV quality is not always high. To CNN-based detectors, image quality is an important part to consider [31], and most state-of-the-art object detection methods take high-quality images as input for granted [22,23,24,25,32,33]. They usually train and test their models on two widely used open datasets, PASCAL [34] or COCO [35], with plentiful categories for evaluation. However, the resolutions of images in the above datasets are usually much higher than those captured from existing governmental CCTV systems that have been established for over 10 years, as used in this work. To deal with the low-resolution impact on vehicle detection accuracy, the low-resolution images (352 × 240) captured from CCTVs were resized to 608 × 608 as the input of CNN to extract more features.

**Figure 2:**
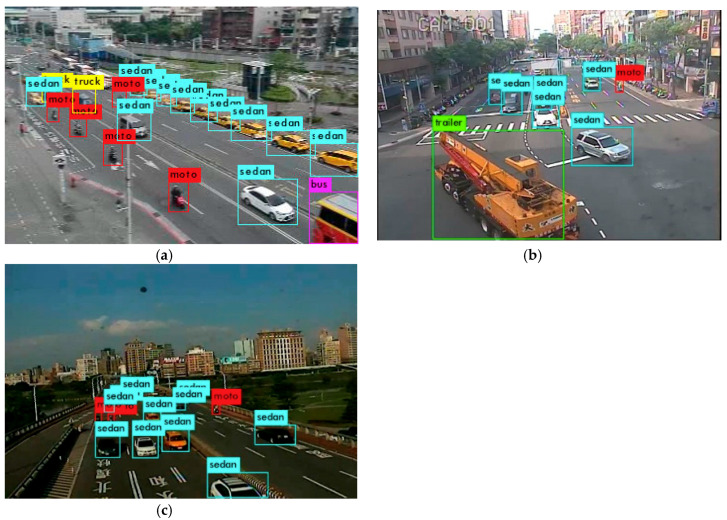
Detection results on (**a**) the road, (**b**) the crossroad, and (**c**) the bridge. Each vehicle is positioned by a colored bounding box. Different colors denote different categories. Purple, yellow, cyan, red, and green represent bus, truck, sedan, motorcycle, and trailer, respectively.

In the training phase, 1951 images were trained by using the YOLOv3-tiny-3l model with a 608 × 608 pixels’ image size. Vehicles were divided into five categories: sedan, motorcycle, bus, truck, and trailer. Multi-scale training was also applied to make the network more adaptable to different sizes of objects. In the analyzing phase, a multi-processing technique was utilized to process each video stream concurrently. At the beginning of each process, images were fetched from the CCTV camera through the internet. The well-trained YOLOv3-tiny-3l model then predicted the positions and types of each vehicle in the image. Figure 2 shows some detection results under different traffic scenarios. For further traffic information extraction, the same vehicles between the previous image and the current image needed to be tracked and identified. We used the optical flow technique to track the feature points inside the vehicle area. The optical flow method matches the local features of the target objects in the previous image with those of the candidate objects in the next image to find the correspondence between objects between frames [36]. Then, the object movement between frames can be used as a reference for calculating the direction and speed of the vehicle.

**Figure 3:**
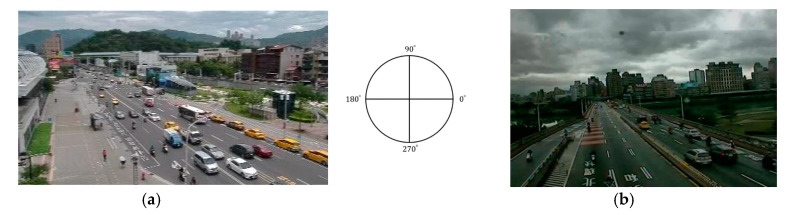
Two examples of defining the traffic directions based on the traffic flow in the traffic lanes near the camera: (**a**) the range of the incoming direction is defined from 140° to 170°, and that of the outgoing direction is defined from 290° to 340°; (**b**) the range of incoming and outgoing directions are defined from 130° to 160° and from 230° to 320°, respectively.

However, it was still a huge challenge to count the vehicles with the same moving direction since the roads and fields of view were different in different CCTV videos. Before analyzing traffic information, we needed to manually define the directions of traffic flow of each CCTV once. Since the cameras’ fields of view were not restricted, the actual geographic direction on the screen could not be confirmed without acquiring camera-related parameters. The expected range of each traffic flow in different CCTVs was predefined in the counterclockwise direction from 0° to 360° according to the traffic flow in the traffic lanes near the cameras. Two examples with different incoming and outgoing traffic directions are shown in Figure 3. The incoming and outgoing traffic directions in the left panel are defined as 140–170° and 290–340°, and those in the right panel are defined as 130–160° and 230–320°, respectively. We counted the number of “tracked” vehicles and estimated the average speed for the longer object trajectories toward a specific direction every 5 min to reduce the error caused by the poor quality of streaming video and the perspective distortion of object trajectories. On the other hand, we also counted the number of “detected” vehicles to estimate the average number of vehicles on roads every 5 min. This value is usually high in the case of a traffic jam, while the value of throughput is low or even zero in off-peak periods.

To evaluate the detection accuracy of the system, we took 20% of the labeled data, about 488 images, to calculate the precision and recall rates, with the true positive (TP), the false positive (FP), and false negative (FN) of each category as follows:

Precision = TP/(TP + FP),(1)

Recall = TP/(TP + FN),(2)

If a vehicle was detected and classified correctly, it was counted as a TP. On the contrary, if a vehicle was falsely detected or classified incorrectly, it was counted as an FP.

### 2.3. PM_2.5_ Sensing and Vehicle Counting in Fieldwork

The LCS devices used in this work are AS-LUNG-O, integrated by our team [13]. AS stands for Academia Sinica, the research institute supporting its development; LUNG indicates the human organ most commonly affected by air pollutants; and O indicates the “outdoor” version. AS-LUNG-O (≈650 USD basic manufacturing cost) incorporates sensors for PM_2.5_ (PMS3003, Plantower, Beijing, China), CO_2_ (S8, Senseair AB, Delsbo, Sweden), temperature/humidity (SHT31, SENSIRION, Staefa ZH, Switzerland), and Global Positioning System (GPS, u-blox, Thalwil, Switzerland). The sensors are placed in a waterproof shelter connected to a solar panel with backup batteries for the power supply, with the option of using household electricity where easily accessible. The size of the whole set is roughly 60 (W) × 50 (D) × 50 cm (H), with a weight of approximately 4.8 kg. Data can be transmitted wirelessly by the built-in 4G modules to a cloud database. An SD card was added as a complement to avoid data loss during wireless transmission. The data correction of AS-LUNG-O with a research-grade instrument, GRIMM 1.109 (GRIMM Aerosol Technik GmbH & Co. KG, Ainring, Germany), and its application for community source evaluations were presented in a previous paper [13]. The data from GRIMM 1.109 also had excellent agreement (R^2^ = 0.999, with roughly ±11% bias) with data from an EDM-180 (GRIMM Aerosol Technik Ainring GmbH & Co., Ainring, Germany) [21], an FEM instrument designated by the USEPA for PM_2.5_.

For the fieldwork to collect actual PM_2.5_ and vehicle counts, the locations of governmental CCTV cameras for traffic analysis were first identified for Taipei and New Taipei cities, two neighboring metropolises in northern Taiwan. A site survey was carried out to assess the locations suitable to set up AS-LUNG-O sets near the cameras. The ideal locations for AS-LUNG-O were in the same block and on the same side of the traffic flow aimed at by cameras. The images used for vehicle detection in this work came from two roadside AS-LUNG-O locations, with the one in Taipei City marked as Location A and the other in New Taipei City marked as Location B. One AS-LUNG-O has been attached to a light pole at 2.5 m above ground at each roadside location since 2017.

**Figure 4:**
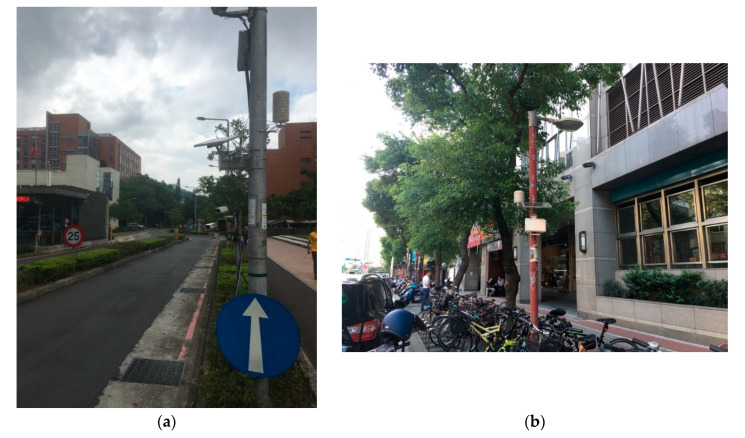
Site photos for AS-LUNG-O sets at (**a**) Location A and (**b**) Location B.

Location A was at the roadside of the main entrance (Figure 4a) of a research institute with two entrance lanes (in the photo) and two exit lanes. No motorcycles are allowed to enter. Thus, the major vehicles entering are sedans, with very limited numbers of buses, trucks, and trailers. The videos of the traffic entering and exiting were saved on disks and analyzed for vehicle counts afterwards. In addition, another AS-LUNG-O assessing background PM_2.5_ without vehicle emission was set up at 2.5 m above ground inside the campus of this institute, with few vehicles passing by, within 200 m from Location A. A HOBO weather station (HOBO RX3000, Onset Computer Corporation, Bourne, MA, USA) was set up on the rooftop (10 m above ground) of a nearby building of the background location to acquire meteorological observations. This research institute can be viewed as a closed community, with the majority of vehicles using the main entrance.

Location B was located at the roadside of a six-lane street in the periphery of a residential community, which was an open community with several streets passing through the community or in the surroundings. Videos from one camera in Location B were analyzed in real time by on-line streaming analysis for vehicle counts in both directions of the traffic. Moreover, in order to assess background PM_2.5_ levels without direct vehicle emission in the same community at the same time, one AS-LUNG-O was set up on the rooftop of an elementary school (15 m above ground) within 200 m of Location B. An aforementioned HOBO weather station was set up at this background location to acquire meteorological observations.

The data captured during rainy hours were excluded from the dataset since rain washes out PM_2.5_. PM_2.5_ observations with 1-min resolutions from AS-LUNG-O were converted to GRIMM-comparable measurements according to correction equations (R^2^ = 0.978–0.991) obtained from the side-by-side laboratory evaluations with GRIMM described earlier [13]. The vehicle counts in lanes near the AS-LUNG-O sets were denoted as “near”, such as sedan-near, while those in lanes of the opposite direction were denoted as “far”, such as sedan-far. After trial and error, the images used in this work were from April 2018 to August 2019 at Location A and May–December 2019 at Location B.

### 2.4. Data Fusion for PM_2.5_ Concentration Increments

Environmental sensing of PM_2.5_ levels and deep learning on vehicle classification and counting were used to acquire roadside PM_2.5_ concentration increments. GRIMM-comparable PM_2.5_ measurements from AS-LUNG-O and vehicle counts for five different vehicle types were matched with a 5-min resolution. Since only a few vehicles passed by between 10 pm and 7 am, only vehicle counts from 7 am to 10 pm were kept in the dataset. Afterwards, regression models were established to assess the incremental contribution of each vehicle of different vehicle types to roadside PM_2.5_ levels, according to similar concepts in previous studies [13].

PM_roadside_ = β0 + γ1 PM_background_ + γ2 temperature + γ3 RH + γ4 Ws + ∑ βi Xi + ε (3)

where PM_roadside_ is 5-min PM_2.5_ at the roadside monitoring location, and PM_background_ is 5-min PM_2.5_ at the background site without direct vehicle emission and within a 200 m radius of the location of PM_roadside_; β0 is the intercept; γ1, γ2, γ3, γ4, and βi are regression coefficients; Ws is the wind speed (from HOBO weather stations at the background locations); ε is an error term. PM_background_ is used to adjust for day-to-day variations in PM_2.5_, which are affected by other regional sources. The selected background site near Location A had only few vehicles passing by every day. Therefore, the measured levels at the background site could be regarded as the general condition of PM_2.5_ levels in the air. Xi is a variable of weighted vehicle counts of different vehicle types. According to the distance from the center of the lanes that are “near” and “far” from the AS-LUNG-O sets (roughly 1:3), the vehicle counts in the near and far categories are weighted by nine to one in the final regression analysis. Theoretically, Xi should include vehicle counts of sedans, motorcycles, buses, trucks, and trailers. However, due to limited counts of motorcycles, buses, trucks, and trailers at two roadside monitoring locations, these four categories were combined together as “others” in the regression analysis.

## 3. Results

This section presents the results of evaluation of a deep-learning traffic analysis system, roadside PM_2.5_ sensing, and the PM_2.5_ increment estimations based on the fusion of traffic counts and PM_2.5_ measurements.

### 3.1. Evaluation of Traffic Analysis System

Twenty percent of the 2439 labeled images were used to evaluate the performance of YOLOv3-tiny-3l. The total number of TPs and FPs is 3887 and 560, respectively, and the total number of missed detections (i.e., FN) is 726. The precision rate is 87% and the recall rate is 84% (Table 1). Although the detection accuracy was more or less dependent on some uncontrollable factors, such as camera shooting angles, network quality, and weather, the proposed system could still achieve an 87% precision rate for the detection in the daytime without heavy rain. In addition, the computer resources required for this software are not very expensive. For on-line stream analysis, roughly 800 MB GPU memory is needed for each streaming CCTV video. Through a multi-process technique, up to 12 videos can be analyzed simultaneously using a single Nvidia GTX1080ti GPU with 11 GB. In terms of storage space, each camera only generates 80 MB of traffic data for a month.

Governmental cameras come in a wide variety of resolutions from 352 × 240, 640 × 480, 960 × 480 pixels and even larger. It is impractical to train the model corresponding to each resolution. In order to train a model that was sufficient for most resolutions, we used bilinear interpolation to scale the input image size to 608 × 608 pixels, which was still real time in inference phase and affordable for training on consumer-grade GPU. From current experience, enlarging the image size before providing them to CNN helps improving the accuracy [28,37,38], and our experiment also confirms this result. Our results showed that the precision and recall rates were 0.85 and 0.79, 0.87 and 0.83, and 0.87 and 0.84 for the image sizes of 416 × 416, 512 × 512, and 608 × 608 pixels, respectively.

Moreover, YOLOv4 was applied to the same images for comparison with YOLOv3-tiny-3l. The precision and recall rates of YOLOv4 with 416 × 416 pixels were slightly better than those of YOLOv3-tiny-3l with 608 × 608 pixels (Table 1). As the size of the image increased to 512 × 512 pixels, the overall performance was further improved. Compared with the result of YOLOv3-tiny-3l, the total number of TPs of YOLOv4 was higher. Meanwhile, the total number of false detections and missed detections were both decreased. YOLOv4 could especially improve the detection rate to a great extent. While the precision rate increased only slightly, there was a 6–7% increase in the recall rate. This suggests the potentially more accurate performance of YOLOv4 to detect target objects. In terms of detection speed, YOLOv4 was capable of carrying out the real-time detection task. The detection speeds of YOLOv4 with an input image size of 416 × 416 and 512 × 512 were 42 and 35 fps, respectively. The only issue of concern is the GPU memory consumption. YOLOv3-tiny-3l needs roughly 800 MB GPU memory to process data from a CCTV camera; the GPU memory requirement in YOLOv4 is 2.5–3 times that needed by YOLOv3-tiny-3l. In other words, the number of CCTV cameras that could be simultaneously processed is reduced to one-half to one-third with YOLOv4. YOLOv4 was published after our team had finished the field campaigns; thus, the following traffic analysis was carried out with YOLOv3-tiny-3l. As shown in Table 1, YOLOv4 with the image size of 416 × 416 pixels reached higher precision and recall rates than YOLOv3-tiny-3l with the image size of 608 × 608 pixels. Furthermore, we also enlarged the image size to 512 × 512 pixels and found that there was no significant improvement in precision or recall rates. For more testing, we tried to rescale the image size to 608 × 608 pixels. However, due to the GPU memory limitation, we failed to train the YOLOv4 model with the image size of 608 × 608 pixels.

### 3.2. PM_2.5_ Sensing and Vehicle Counting

Table 2 shows the summary of the results of PM_2.5_ sensing and vehicle counting of different vehicle types in two roadside monitoring locations with a 5-min resolution. The roadside PM_2.5_ levels at Locations A (17.6 ± 9.2 µg/m^3^) and B (16.5 ± 6.8 µg/m^3^) are close to each other. The wind speeds measured near Location A (0.73 ± 0.73 m/s) seem to be much lower than those near Location B (2.55 ± 1.05 m/s). For vehicle counts, the numbers of sedans at Location B, with an average of 45 sedans every 5 min, are four times higher than those at Location A, with an average of 10 sedans every 5 min. It was expected that traffic near Location B would be much busier since it is part of the city traffic, while the traffic near Location A is associated with the research institute only. The numbers of motorcycles, buses, trucks, and trailers are small at both locations; therefore, these are combined in the following regression analysis in the “others” category. Moreover, average driving speed was calculated after removing the samples without any vehicles in that 5-min period in order to estimate the average driving speeds of the passing-by vehicles. For Location A with vehicles entering and for Location B in both directions, the average driving speeds were around 33–44 km/h, while for vehicles leaving the research institute, the average driving speeds became much higher (52.7 ± 19.7 km/h).

### 3.3. Incremental PM_2.5_ Concentration Increase due to Vehicles

The results of the multiple regression are shown in Table 3, with all estimated coefficients with statistical significance at the *p* = 0.001 level. The R^2^ values at Locations A and B were 0.983 and 0.612, respectively. After adjusting for the day-to-day PM_2.5_ variations (background PM_2.5_) and environmental factors (temperature, humidity, and wind speed), the incremental PM_2.5_ concentration increases at the roadside due to one sedan could be quantified as 0.0027 and 0.0050 µg/m^3^ with a 5-min resolution at Locations A and B, respectively. For other vehicle types, the coefficient at Location A was not statistically significant, while that at Location B was negative. This is possibly due to the fact that the numbers of other vehicles at Location B were, on average, only 11–13 (Table 2). This may indicate that this method is only applicable for high traffic counts.

## 4. Discussion

### 4.1. Vehicle Classification/Counting System

This work applied a traffic analysis system to assess vehicle counts of different vehicle types, including sedans, motorcycles, buses, trucks, and trailers. With 488 images from 23 governmental cameras with low resolutions of 352 × 240 pixels, the precision and recall rates of YOLOv3-tiny-3l are 87% and 84%, respectively. As evaluated earlier [27], in terms of the trade-off between the display speed and accuracy, YOLOv3 with resolutions of 320, 416, and 608 was overall better than SSD321, SSD513, DSSD321, DSSD513, R-FCN, FPN FRCN, RetinaNet-50-500, Retina-101-500, and RetinaNet-101-800. However, these systems all require expensive computer systems. YOLOv3-tiny-3l is a lightweight version of YOLOv3; it can operate with a consumer-grade GPU. Thus, it is an inexpensive alternative object detection system capable of analyzing vehicle counts. Its precision rate is close to that of the newest version YOLOv4, but its recall rate is obviously not as good as those from YOLOv4. Nevertheless, since the aim of our work was to assess vehicle counts in real time with multiple inputs, the size of YOLOv4 would have restrained the numbers of CCTV cameras that could be processed simultaneously. Therefore, for our purpose, YOLOv3-tiny-3l is the best choice to conduct a real-time assessment of vehicle counts of different vehicle types.

Besides using object detection techniques, license-plate recognition techniques were also used by other research groups to extract vehicle information, e.g., [39]. The license-plate recognition scheme needs to access a governmental database to distinguish whether the vehicle is a sedan, truck, or trailer. Partial blocking of license plates by other objects would affect the accuracy of the recognition [39]. For public goods, such as law enforcement or intelligent transportation management, governmental authorities have the right to access databases for extracting detailed vehicle information by recognizing license plates. However, ordinary researchers do not have access to governmental databases for license-plate recognition, which may also be a concern of invasion of privacy. Therefore, the object detection of the shape of the vehicles, as used in this work, is a better alternative for research applications.

### 4.2. Incremental Contribution of PM_2.5_ Levels at Roadsides of Vehicles

This work utilized data fusion of environmental sensing of PM_2.5_ and deep learning on vehicle classification/counting to evaluate incremental PM_2.5_ contributions of each passing-by sedan to roadside PM_2.5_ levels. Most traffic emission studies have focused on the contribution of traffic emission to city-wide pollutant levels, which is important for source apportionment and control strategy prioritization. Nevertheless, it is also essential to understand the traffic contribution to pedestrian exposure levels, which may lead to subsequent health impacts. The direct emission of vehicles results in close and peak exposures that cannot be captured by the EPA monitoring stations situated at 10–15 m above ground. With calibrated PM_2.5_ LCSs, we are able to assess roadside PM_2.5_ levels. Our previous work quantified the contribution of vehicle emissions to roadside PM_2.5_ levels [13]; it was found that stop-and-go traffic and passing-by vehicles on average contributed to roadside PM_2.5_ levels of 4.38 and 3.31 µg/m^3^, respectively. However, no detailed vehicle counts or types were available at that time. Thus, we could not quantify the contribution of each vehicle previously. The current work takes advantage of a traffic analysis system based on deep learning and traditional optical flow techniques to count the numbers of vehicles with five different types. As a result, we can quantify the incremental contribution of each sedan to roadside PM_2.5_ levels. Unfortunately, the contributions of other types of vehicles were either not statistically significant or contrary to our intuition due to the small sample size. More data are needed for further discussion.

The incremental contributions of each sedan to roadside PM_2.5_ levels at Locations A and B were estimated to be 0.0027 and 0.0050 µg/m^3^ with a 5-min resolution, respectively. This estimation was obtained based on weighted vehicle counts, with the vehicle counts in traffic lanes near AS-LUNG-O weighted as 90% and the vehicle counts in traffic lanes in the opposite direction weighted as 10%. The on-average contribution of each sedan to roadside PM_2.5_ at Location A was lower than that at Location B, possibly due to the slightly slower speed (33.2 ± 11.9 km/h) of vehicles entering the institute compared with the speed of vehicles near Location B (43.8 ± 14.3 km/h in traffic lanes near the camera). Although the driving speed of vehicles leaving the institute was much higher (52.7 ± 19.7 km/h), the impact of the driving speeds of vehicle counts of exiting lanes was smaller than that of the entrance lanes due to the farther distance.

The estimation of the incremental contribution of each sedan (or other types of vehicles) is important for comparing the extent of traffic regulation enforcement among different areas. For example, in Taiwan, the central government sets traffic emission standards for each type of vehicle, and local/city governments are in charge of inspecting the compliance of on-road vehicles [40,41]. Some local governments may not implement the enforcement strictly, resulting in significant pollutant emissions of on-road vehicles. With our methodology to assess roadside concentration increments for each sedan in different areas, citizens will be informed of actual pedestrians’ PM_2.5_ exposures due to on-road vehicles in that area. Since Taiwan citizens are concerned about carcinogenic PM_2.5_ levels, the information on exposure increments due to on-road vehicles will put pressure on local/city governments to enhance the extent of law enforcement to reduce vehicle tailpipe emissions.

Furthermore, traffic emissions may also have impacts on the indoor air quality of the buildings along the streets. Asian cities usually have high population densities, and residences are packed along the busy streets. Our previous work found that 12.3% of residents in the Taipei metropolitan area actually live on the first or second floor within 5 m of municipal roads [42]. Infiltration of vehicle exhaust into indoor environments was also demonstrated by other researchers who found traffic-related elements indoors [43]. The roadside PM_2.5_ at Location B may disperse further to affect indoor PM_2.5_ levels in the residential buildings on that street via air exchange and impact the residents’ PM_2.5_ exposures in households. With LCSs, the impacts of traffic on indoor air quality can also be assessed in the future.

Our monitoring of PM_2.5_ and vehicle counts has been ongoing since 2017. However, only the observations taken during the periods of April 2018 to August 2019 at Location A and May–December 2019 at Location B were included in the above analysis. This is because the images of the governmental cameras in other periods had different view angles from those of the training sets. The governmental cameras used for traffic monitoring were controlled by governmental officials who sometimes turned cameras to face a different direction. Sometimes the cameras returned to their original directions, and sometimes they did not. Thus, image processing could not proceed since the image was different from those of the training sets. This was the biggest challenge of our work. We actually needed to save one image per hour to keep track of whether the direction of the camera was changed. Only the images with the same orientation as the training set were included in the final data analysis. Thus, it took a while to accumulate enough samples for our analysis. An alternative to avoid this interference would be setting up our own cameras in streets for vehicle counting, which requires on-site electricity and maintenance. In fact, we installed AS-LUNG-O sets in five different locations near the governmental cameras. However, the cameras in the other three locations turned too frequently to be used for our analysis. Thus, only images of certain periods at Locations A and B were used in the current work. There are no perfect solutions for dealing with real-world situations. In this case, we chose to use governmental cameras that have already been installed in hundreds of different locations, and the images needed to be checked regularly to determine whether the directions were the same as those of the training sets. For researchers in other countries using similar methods, the stability of camera orientation is an important factor to consider.

Moreover, in the current emission inventory, pollutant emission factors were obtained in a laboratory on the basis of a limited quantity of vehicles under controlled conditions; significant discrepancies have been found between emission factors based on laboratory tests and those from on-road measurements due to various factors [12,44]. The actual driving conditions may be outside the range of the controlled conditions in the laboratory; the fuel used, the maintenance, and the drivers’ driving habits can affect the actual emissions of in-use vehicles [12]. These uncertainties lead to even greater ambiguity in the subsequent air quality modeling results and pollution control strategies, which may compromise the effectiveness of air pollution reduction policies. Moreover, the 2015 Volkswagen scandal showed that the automotive industry purposely cheated in laboratory tests; the actual on-road pollutant emissions were much higher than those presented in the official laboratory tests [45,46]. This bias has resulted in appreciable health damages [47,48]. The aforementioned facts raise the need to investigate the on-road emission factors of in-use vehicles. Several techniques, such as remote sensing, road tunnel studies, and portable emission measurement systems, have been used to obtain the on-road emission factors of vehicles; they all have certain advantages and limitations [12]. The current work applies an LCS device for PM_2.5_ monitoring and deep learning techniques for vehicle detection to acquire roadside PM_2.5_ concentration increments due to vehicles in real-world situations. For the accumulation of such estimates to obtain a large sample size, on-road PM_2.5_ emission factors may be derived in combination with street canyon modeling [49], providing an innovative alternative for acquiring vehicle emission factors. This requires further analysis. Street canyon models typically use pollutant emission factors to simulate roadside concentration increments [49]. The inverse modeling concept could be used in street canyon models to derive emission factors based on roadside PM_2.5_ concentration increments.

The main purpose of this manuscript is to demonstrate the applicability of this data fusion methodology. Originally, it was intended to quantify the contribution of five different types of vehicles for comparison. However, it turned out that the number of the other types of vehicles passing by these two roadside locations was not large enough to be analyzed. This is a pioneer work. We did not know that this method works only in the presence of a larger number of vehicles before our data analysis. The estimated coefficient for each sedan from the regression analysis was based on large vehicle counts of sedans per 5 min. For the single type vehicle “sedan”, both mean values of “near” and “far” vehicle counts were higher than 10 (Table 2). We weighted vehicle counts of “near” and “far” traffic flows by 0.9 and 0.1, respectively. Thus, the weighted sum of “near” and “far” vehicle counts for sedans was 10.4 at Location A and even higher at Location B. The estimated coefficients were statistically significant with positive PM_2.5_ contributions. However, for mixed types of vehicle counts at Location B, the sum of the mean vehicle counts for other types of vehicles (“others” in Table 3) at “near” was 7.9 + 1.1 +2.1 = 11.1 and 9.1 + 2.0 + 2.3 = 13.4 at “far”. The weighted sum of “near” and “far” vehicle counts was 11.3 (11.1 × 0.9 + 13.4 × 0.1), which was higher than that of single type vehicle. The result of “others” at Location B had negative contributions, in contrast to our common sense. Therefore, we suspected that this method may not work for vehicle counts less than 11–13 (the sums of mean vehicle counts for “others” at “near” and “far” were 11.1 and 13.4, respectively) of multiple types. There are certain streets in Taiwan with designated motorcycle lanes and truck lanes. In the future, we can identify such locations with governmental cameras and install AS-LUNG-O in nearby light poles to conduct similar works to assess the contribution of motorcycles and trucks. Nevertheless, this manuscript shows that this method is applicable in the real world. The contributions of vehicles to pedestrians’ exposures were capable of being quantified for each sedan. This method can be used in other countries to evaluate exposure contributions of vehicles as well.

### 4.3. Limitation of This Work

There are several limitations of this work. First, YOLOv3-tiny-3l may not be effective in vehicle counting during raining periods or at night when images are not well-lit. Fortunately, this did not affect our analysis since raining washed out PM_2.5_, and there were few vehicles in the streets at night. Thus, our analysis’ exclusion of data captured in rainy and nighttime periods does not affect the validity of our findings. Secondly, due to the interference of the camera changing directions, as discussed previously, our analysis was conducted for only two locations. In addition, wind at street levels is turbulent so that air pollutants are forced to be mixed well. However, a strong prevalent wind may affect the results of current estimates. This limitation could be tackled by considering the effect of wind direction in the estimation of incremental contributions in future works. Analysis for more locations in one city may be required to obtain representative results for roadside PM_2.5_ concentration increments. Nevertheless, this work demonstrates our methodology, which could be carried out in other locations later. Finally, this method is only applicable when the numbers of vehicles passing by are high. For less than 11–13 vehicles of multiple types passing in 5 min, this method may not provide valid results. In the future, we will try to collect data in areas that may have large vehicle counts for multiple types to evaluate this point further. More evaluation needs to be conducted to assess the threshold of traffic counts for this fusing methodology to validly assess roadside PM_2.5_ concentration increments.

## 5. Conclusions

This work demonstrates the applicability of a data fusion methodology by using PM_2.5_ levels obtained by LCSs and vehicle counts based on deep learning to assess roadside PM_2.5_ concentration increments due to vehicles. YOLOv3-tiny-3l is shown to be a useful tool to assess vehicle counts of sedans, motorcycles, buses, trucks, and trailers as well as vehicle speeds in real time from low-resolution governmental cameras with only 352 × 240 pixels. In addition, roadside PM_2.5_ levels were assessed with LCS devices designed for long-term outdoor PM_2.5_ monitoring, AS-LUNG-O sets, installed in light poles for nearly 8–13 months. It was estimated that roadside PM_2.5_ concentration increments due to each on-road sedan were on average 0.0027–0.0050 µg/m^3^ when the average driving speed was around 30–40 km/h. With the advancement of sensor technology and data science, environmental researchers are able to assess close-to-reality PM_2.5_ exposure estimates, which could be a basis for subsequent health risk assessment or source control prioritization. Compared with traditional expensive PM_2.5_ instruments and vehicle counting systems, our method has great potential to be applied in developing countries to assess the exposure contribution of vehicle emissions.

## Figures and Tables

**Table 1:** The counts of different vehicle types based on 2439 images with YOLOv3-tiny-3l (608 × 608), YOLOv4 (416 × 416), and YOLOv4 (512 × 512), as well as the resultant precision and recall rates.

Detector	Category	Sedan	Motorcycle	Bus	Truck	Trailer	Precision	Recall
YOLOv3-tiny-3l	True positive (TP)	2233	1335	151	143	25		
(608 × 608)	False positive (FP)	259	236	14	47	4		
	False negative (FN)	262	358	27	70	9	87%	84%
YOLOv4	TP	2332	1455	165	175	27		
(416 × 416)	FP	249	259	16	37	6		
	FN	163	238	13	38	7	88%	90%
YOLOv4	TP	2337	1483	166	169	28		
(512 × 512)	FP	255	241	14	27	7		
	FN	158	210	12	44	6	88%	91%

**Table 2:** Distribution of PM_2.5_, environmental factors, and counts of different vehicle types at two roadside monitoring locations in 5-min resolutions: “near” indicates vehicle counts and speed in the traffic lines close to the AS-LUNG-O, and “far” indicates those in traffic lines in the opposite directions.

	Location A (*n* = 33,922)		Location B (*n* = 26,729)	
	Mean	SD ^2^	Mean	SD
Roadside PM_2.5_ (µg/m^3^)	17.6	9.2	16.5	6.8
Temperature (°C)	28.5	5.1	28.1	4.52
RH (%)	71.9	12.2	59.1	11.0
Wind speed (m/s)	0.73	0.73	2.55	1.05
Background PM_2.5_ (µg/m^3^)	17.2	9.1	10.8	3.9
Sedan_near	10.4	8.5	45.4	31.4
Motocycle_near	NA	NA	7.9	9.1
Bus_near	0.04	0.20	1.1	1.4
Truck_near	0.50	0.87	2.1	2.5
Trailer_near	0.00	0.00	0.00	0.03
Speed_near ^1^ (km/h)	33.2 (*n* = 42,243)	11.9	43.8 (*n* = 25,710)	14.3
Sedan_far	10.2	8.01	43.2	27.6
Motocycle_far	NA	NA	9.1	9.3
Bus_far	0.04	0.25	2.0	1.9
Truck_far	0.58	1.1	2.3	2.7
Trailer_far	0.00	0.01	0.00	0.03
Speed_far ^1^ (km/h)	52.7 (*n* = 39,546)	19.7	35.0 (*n* = 26,242)	12.0

^1^ Speed was calculated when the numbers of vehicles in the corresponding direction were above zero; thus, sample sizes were different from those of the others. ^2^ SD: standard deviation; NA: not applicable.

**Table 3:** Incremental PM_2.5_ contributions (µg/m^3^) of various factors from multiple regression analysis at two roadside locations.

	Location A		Location B	
	Coefficient	SE	Coefficient	SE
Intercept	4.15 *	0.096	2.54 *	0.438
Background PM_2.5_ (µg/m^3^)	0.99 *	0.001	1.16 *	0.007
Temperature (°C)	−0.13 *	0.002	−0.186 *	0.009
RH (%)	0.0004	0.001	0.147 *	0.004
Wind speed (m/s)	0.0095	0.01	−0.60 *	0.026
Sedan (count)	0.0027 *	0.001	0.0050 *	0.001
Others (count)	NA	NA	−0.039 *	0.002
R^2^	0.983		0.612	

* *p* < 0.001; SE: standard error; NA: not available.
